# Long-Range Planning for Group Home Dementia Care for Adults with Intellectual Disability

**DOI:** 10.1093/geroni/igaf122.1840

**Published:** 2025-12-31

**Authors:** Matthew Janicki

**Affiliations:** University of Illinois Chicago, Rockport, Maine, United States

## Abstract

With the increasing prevalence of dementia among adults with intellectual disability (ID), there is a growing need for specialized, sustainable community-care models. Community-based group homes offer an alternative to institutional settings, providing trained staff, dementia-friendly environments, and individualized support. However, long-term planning is crucial for resource allocation, service intensity, and continuity of care. This longitudinal study (2011–present) examined the operation of three 5-person dementia-capable group homes, tracking 15 adults with ID/dementia and 15 matched non-dementia controls. At entry, five participants had Down syndrome, nine were male, and their mean age was 59.1 years. The dementia cohort exhibited higher mortality, with one survivor compared to five in the control group. Over 14 years, 35 adults cycled through the homes, with an average length of stay (LOS) of 4.75 years prior to death. LOS was shorter for older adults with complex health conditions. Admission age patterns revealed a tri-modal distribution (50.5, 57.0, and 66.5 years), influencing care needs and longevity. Comorbidities increased over time (8.6 at entry to 12.1 at death), with fluctuations per home based on resident turnover. A key management strategy involved grouping residents by dementia stage, with one home dedicated to advanced dementia and the other two generally similar as to care needs. These findings highlight the importance of age at admission, dementia stage, morbidity trajectories, and mortality-driven transitions in long-term planning. By integrating these factors, providers can optimize staffing ratios, enhance care quality, and ensure the sustainability of dementia-capable group homes for adults with ID.

